# Exploring User Experience and the Therapeutic Relationship of Short-Term Avatar-Based Psychotherapy: Qualitative Pilot Study

**DOI:** 10.2196/66158

**Published:** 2025-02-05

**Authors:** Byeul Jang, Chisung Yuh, Hyeri Lee, Yu-Bin Shin, Heon-Jeong Lee, Eun Kyoung Kang, Jeongyun Heo, Chul-Hyun Cho

**Affiliations:** 1 Department of Psychiatry, Korea University College of Medicine, 73 Goryeodae-ro, Seongbuk-gu, Seoul, 02841, Republic of Korea, 82 029205505; 2 Department of Medical Education, Korea University College of Medicine, Seoul, Republic of Korea; 3 Annenberg School for Communication and Journalism, University of Southern California, Los Angeles, CA, United States; 4 KakaoHealthcare Corp., Seongnam, Republic of Korea; 5 Department of Smart Experience Design, Kookmin University, Seoul, Republic of Korea; 6 Department of Biomedical Informatics, Korea University College of Medicine, Seoul, Republic of Korea

**Keywords:** avatar-based psychotherapy, telehealth, therapeutic relationship, user experience, anonymity, nonverbal communication, mental health, mobile phone

## Abstract

**Background:**

The rapid advancement of telehealth has led to the emergence of avatar-based psychotherapy (ABP), which combines the benefits of anonymity with nonverbal communication. With the adoption of remote mental health services, understanding the efficacy and user experience of ABP has become increasingly important.

**Objective:**

This study aimed to explore the user experience and therapeutic relationship formation in short-term ABP environments, focusing on psychological effects, user satisfaction, and critical factors for implementation.

**Methods:**

This qualitative study involved 18 adult participants (8 women and 10 men). Participants engaged in two short-term ABP sessions (approximately 50 minutes per session) over 2 weeks, using an ABP metaverse system prototype. Semistructured in-depth interviews were conducted with both the participants and therapists before and after the ABP sessions. The interviews were conducted via an online platform, with each interview lasting approximately 30 minutes. The key topics included the sense of intimacy, communication effectiveness of avatar expressions, emotions toward one’s avatar, concentration during sessions, and perceived important aspects of the ABP. Data were analyzed using thematic analysis.

**Results:**

The analysis revealed 3 main themes with 8 subthemes: (1) reduction of psychological barriers through avatar use (subthemes: anonymity, ease of access, self-objectification, and potential for self-disclosure); (2) importance of the avatar–self-connection in therapeutic relationship formation (subthemes: avatar self-relevance and avatar–self-connection fostering intimacy and trust); and (3) importance of nonverbal communication (subthemes: significance of nonverbal expressions and formation of empathy and trust through nonverbal expressions). Participants reported enhanced comfort and self-disclosure owing to the anonymity provided by avatars, while emphasizing the importance of avatar customization and the role of nonverbal cues in facilitating communication and building rapport.

**Conclusions:**

This pilot study provides valuable insights into the short-term ABP user experience and therapeutic relationship formation. Our findings suggest that ABP has the potential to reduce barriers to therapy through anonymity, ease of access, and potential for self-disclosure, while allowing for meaningful nonverbal communication. The avatar–self-connection emerged as a crucial factor in the effectiveness of ABP, highlighting the importance of avatar customization in enhancing user engagement and therapeutic outcomes. Future research and development in ABP should focus on improving avatar customization options, enhancing the fidelity of nonverbal cues, and investigating the long-term effectiveness of ABP compared with traditional face-to-face therapy.

## Introduction

Rapid advancements in information technology have led to significant developments in telehealth, particularly in addressing mental health issues, such as anxiety, depression, and stress-related disorders [1]. The COVID-19 pandemic has accelerated the transition from traditional face-to-face clinical services to telehealth solutions [2]. This shift has created new opportunities for individuals with difficulty accessing in-person psychotherapy owing to geographical constraints or personal reservations [3]. Avatar-based psychotherapy (ABP) has gained attention because of its unique ability to combine the benefits of anonymity and nonverbal communication. Recent research has demonstrated the potential effectiveness of metaverse-based counseling approaches compared to traditional in-person settings [4].

The therapeutic relationship between the client and psychotherapist is crucial for successful therapeutic outcomes. However, the dynamics of this relationship in ABP environments require further investigation [5]. Self-disclosure, a key element in relationship building, may be facilitated differently in ABP settings than in traditional face-to-face or video-based online therapy [6]. The concept of avatar–self-connection, which refers to the psychological link between users and their digital representations, may play a significant role in the effectiveness of ABP [7].

This study aimed to explore the user experience and therapeutic relationship formation in short-term ABP environments. Specifically, we sought to understand the psychological effects and satisfaction levels of users engaging in ABP and to identify critical factors to consider when implementing ABP systems.

## Methods

### Ethical Considerations

This study was conducted in accordance with the principles of the Declaration of Helsinki and approved by the Institutional Review Board of Korea University Anam Hospital (IRB No: 2023AN0504). All participants provided written informed consent before participation. Participants were informed that their participation was voluntary and that they could withdraw at any time without consequences. All interviews were audio-recorded with prior consent and subsequently transcribed for analysis. The data collected in this study were anonymized to ensure the privacy and confidentiality of all participants. This manuscript and its supplementary materials do not include any images or identifiable features of research participants.

### Study Design and Data Collection

This study was designed as a short-term intervention pilot study to explore initial user experiences with ABP. The two-session format was chosen to capture immediate reactions and early therapeutic relationship formation in the ABP environment. We conducted a qualitative study to gain an in-depth understanding of user experiences in ABP sessions. Eligible participants were adults aged 19‐70 years who self-reported their need for psychological health management and had no previous experience with avatar-based counseling. We excluded individuals with intellectual disabilities, organic brain damage, or those currently receiving psychiatric treatment for major mental disorders (including major mood disorders, anxiety disorders, and schizophrenia spectrum disorders). Additionally, participants were required to have access to and the ability to use smartphones or computers.

**Figure 1.**
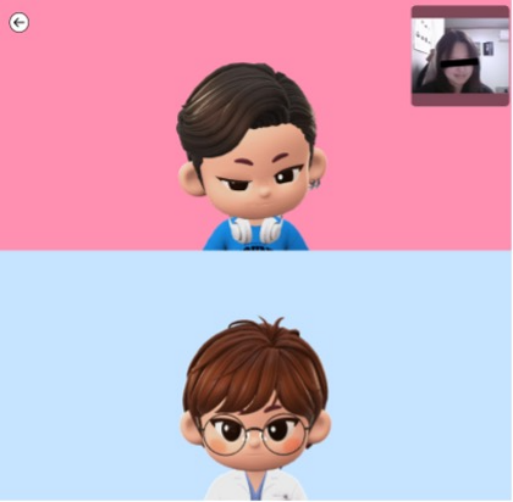
The avatar-based psychotherapy metaverse system prototype. Top avatar: participant; bottom avatar: psychotherapist. The real face box at the top can be turned off; in a real psychotherapy session, the real face is turned off and is conducted with only the avatar.

An avatar that reflected facial movements in real time was established using a metaverse system prototype (Figure 1). This system allowed participants to engage in psychotherapy sessions through avatars, enabling verbal and nonverbal communication. Eighteen adult participants (8 women) were initially recruited in Seoul, South Korea. The participants engaged in two ABP sessions (approximately 50 minutes per session) over 2 weeks.

Semistructured in-depth interviews were conducted with both the participants and psychotherapists before and after the ABP sessions. These interviews, each lasting 30 minutes, were conducted using the Zoom platform (Zoom Video Communications, Inc.). The key topics included the sense of intimacy and communication effectiveness of avatar expressions, emotions and feelings toward one’s avatar, concentration during the session, and perceived important aspects of the ABP sessions.

### Data Analysis

We employed thematic analysis to analyze the data, using ATLAS.ti software (Version 23; ATLAS.ti Scientific Software Development GmbH, Berlin, Germany) [8]. The analysis was conducted in 6 phases: familiarization with the data, generating initial codes, searching for themes, reviewing themes, defining and naming themes, and producing reports. Four researchers (JB, HL, JH, and CHC) participated in the initial familiarization phase and carefully reviewed the transcripts before individual coding. The 174 initial codes were systematically categorized through iterative analysis. Through team discussions, these codes were refined and consolidated into 3 main themes of psychological barriers (41 codes), avatar–self-connection (29 codes), and nonverbal communication (38 codes). The remaining codes were excluded as they did not directly address the research questions.

## Results

The thematic analysis revealed 3 main themes with 8 subthemes, as shown in Table 1. The 174 initial codes were systematically categorized through iterative analysis. Through team discussions, these codes were refined and consolidated into 3 main themes of psychological barriers (41 codes), avatar–self-connection (29 codes), and nonverbal communication (38 codes). The remaining codes were excluded as they did not directly address the research questions.

**Table 1.** Thematic framework of user experiences and therapeutic relationship formation in avatar-based psychotherapy: main themes, subthemes, code counts, and representative codes.

Main themes	Subthemes	Code counts	Representative codes
Reduction of psychological barriers through avatar use	1.1 Anonymity	12	Anonymity reduces social anxiety. Sense of security through anonymity. Honest communication enabled. Anonymity prevents judgment. Reduced fear of personal recognition.
	1.2 Ease of access	10	Flexible scheduling. No travel required. Accessible from anywhere. Easier therapy initiation. Reduced logistical challenges.
	1.3 Self-objectification	8	Detached view encourages self-reflection. Avatar mirrors emotional state. Enhances self-awareness. Observing oneself via the avatar. Identifies overlooked emotions.
	1.4 Potential for self-disclosure	11	Easier to discuss sensitive topics. Safe space for expression. Encourages honest sharing. Enables communication without barriers. Reduces hesitation in disclosure.
Importance of avatar–self-connection	2.1 Avatar self-relevance	15	Personalized avatars foster comfort. Realism enhances connection. Reflects personal identity. Improves immersion. Mirrors user’s traits effectively.
	2.2 Fostering intimacy and trust	14	Facilitates faster rapport. Acts as a conversational bridge. Builds therapeutic trust. Supports meaningful dialogue. Encourages emotional openness.
Importance of nonverbal communication	3.1 Significance of nonverbal expressions	20	Gestures enhance communication. Nonverbal cues reduce misunderstandings. Expressive avatars improve immersion. Adds emotional depth. Reinforces communication effectiveness.
	3.2 Empathy and trust formation	18	Mirrors emotions effectively. Conveys empathy through gestures. Builds trust via nonverbal cues. Enhances emotional understanding. Reduces virtual communication gaps.

### Theme 1: Reduction of Psychological Barriers Through Avatar Use

Theme 1, the reduction of psychological barriers through avatar use, emerged as the most prominent theme with 41 codes. These codes were distributed across 4 subthemes: anonymity (12 codes), ease of access (10 codes), self-objectification (8 codes), and potential for self-disclosure (11 codes). Participants reported that the use of avatars in psychotherapy sessions helped lower psychological barriers primarily through 3 mechanisms.

#### Subtheme 1.1: Anonymity

The anonymity provided by avatars was frequently mentioned as a key factor in facilitating open and honest communication. One participant noted, “Knowing that the therapist does not know my real face gives me a sense of security. Even if we met in real life, they would not recognize me, which is reassuring in a way.”

#### Subtheme 1.2: Ease of Access

The convenience of accessing ABP services through a metaverse platform was highlighted as a significant advantage. One participant stated, “The fact that psychotherapy sessions can be done from anywhere makes it more accessible and easier to form a rapport. I think anonymous ABP is more effective in this regard.”

#### Subtheme 1.3: Self-Objectification

Avatars facilitated self-objectification, allowing participants to view themselves from a detached perspective. One participant observed, “When I occasionally looked at my avatar, I noticed that I was not smiling much. It made me think, ‘I look a bit pitiful.’ This showed me how I appeared.”

#### Subtheme 1.4: Potential for Self-Disclosure

Avatars facilitated self-disclosure. One participant noted, “For people who want psychotherapy without revealing themselves too much, expressing themselves through an avatar in the metaverse can make it easier to talk honestly with the therapist.”

### Theme 2: Importance of Avatar–Self-Connection in Therapeutic Relationship Formation

The importance of avatar–self-connection in therapeutic relationship formation emerged as the second major theme, comprising 29 codes. These were distributed between 2 key subthemes: avatar self-relevance (15 codes) and avatar–self-connection fostering intimacy and trust (14 codes). The results showed that the degree of connection between users and their avatar played a crucial role in the therapeutic relationship formation within the ABP environment.

#### Subtheme 2.1: Avatar Self-Relevance

Participants expressed a desire for their avatars to reflect aspects of their real selves. One participant noted, “If I could customize the avatar more to my liking and feel that it truly represents me, I think I would feel more completely connected to it.”

#### Subtheme 2.2: Avatar–Self-Connection Fostering Intimacy and Trust

A strong avatar–self-connection enhanced the sense of intimacy and trust in the therapeutic relationship. One participant shared, “When I felt that this avatar was really me, I could immerse myself more deeply in the session and form a stronger therapeutic relationship with the therapist.”

### Theme 3: Importance of Nonverbal Communication

The importance of nonverbal communication emerged as a significant theme with 38 codes, distributed across 2 subthemes: significance of nonverbal expressions (20 codes) and formation of empathy and trust through nonverbal expressions (18 codes). The thematic analysis highlighted the significant role of nonverbal communication in ABP, emphasizing its impact on self-expression, empathy, and trust-building.

#### Subtheme 3.1: Significance of Nonverbal Expressions

The participants emphasized the importance of nonverbal cues in enhancing communication. One participant stated, “If the avatar could show hand gestures or body language, I think it would be even better. These physical movements can have psychological significance.”

#### Subtheme 3.2: Formation of Empathy and Trust Through Nonverbal Expressions

Nonverbal communication through avatars enhanced empathy and trust in the therapeutic relationship. One participant shared, “I think the avatar helped by expressing my gestures and expressions to some extent. This somewhat overcomes communication errors that can occur when we are not face-to-face.”

## Discussion

### Study Findings and Comparison With Previous Findings

This study provides critical insights into ABP, highlighting 3 interconnected dimensions: psychological barrier reduction, avatar–self-connection, and the role of nonverbal communication in metaverse therapeutic systems.

The anonymity, accessibility, and potential for self-disclosure provided by avatars create a unique therapeutic environment that lowers the resistance to mental health engagement, consistent with previous research on online self-disclosure [5]. A novel finding of this study was the self-objectification through avatar use, suggesting a potential mechanism for enhanced self-awareness that resonates with self-distancing theories in psychology [9]. This indicates that ABP may offer a natural pathway for adaptive self-reflection, a key component in many therapeutic approaches.

The self-objectification observed in this study demonstrates an interesting contrast with findings from video conferencing research, which found that viewing one’s actual image during video conferences can lead to greater cognitive burden and negative psychological effects, particularly for individuals with high public self-consciousness [10]. However, our findings suggest that avatar-mediated self-observation may offer distinct advantages. Unlike video conferencing where users see their actual image, avatar representation creates a beneficial psychological distance that facilitates more objective self-reflection while maintaining emotional engagement. This unique characteristic of avatar-mediated interaction may explain why participants in our study reported enhanced self-awareness without the negative psychological impacts often associated with direct self-viewing in video conferences. The avatar-mediated environment provides a unique balance between self-awareness and psychological comfort, potentially making it particularly suitable for therapeutic contexts where self-reflection is crucial but emotional safety needs to be maintained.

Furthermore, the avatar–self-connection played a crucial role in the therapeutic relationship formation, extending previous work on avatar realism [7]. Our findings directly linked this connection to the quality of the therapeutic relationship, underscoring the potential significance of avatar customization in fostering engagement and improving outcomes. The avatar may serve as a bridge between the client’s inner world and the therapeutic space, facilitating deeper exploration of personal narratives.

Our observations of nonverbal communication in ABP expand on previous research, highlighting how even limited nonverbal cues can significantly impact the therapeutic process [11]. This adaptability suggests that ABP taps into fundamental aspects of human communication, offering a rich alternative to traditional face-to-face therapy.

The interplay among these elements in ABP creates a unique therapeutic ecosystem that balances self-disclosure and self-protection. As we continue to explore the potential of ABP, it is crucial to consider its place within the broader context of mental health care. ABP can be viewed as a complementary tool that can lead to more comprehensive and accessible care, potentially reaching individuals who might otherwise not receive treatment [12,13].

### Strengths and Limitations

The findings of this study have several implications for clinical practice. First, ABP may be particularly beneficial for clients hesitant to engage in traditional face-to-face therapy because of social anxiety, stigma, or geographical limitations [14]. The anonymity and ease of access provided by ABP could serve as a stepping stone for these individuals to transition to other forms of therapy, if needed. Second, clinicians using ABP should be aware of the importance of the avatar–self-connection and consider ways to enhance this connection during therapy sessions. This might involve discussing the client’s choice of avatar and exploring how it relates to their self-perception or therapeutic goals. However, although ABP offers many advantages, clinicians must be aware of its limitations. The reduced nonverbal cues in ABP compared with face-to-face therapy require clinicians to develop new skills in reading and interpreting client expressions and emotions through avatars.

### Conclusion

These findings not only contribute to the growing body of knowledge on telehealth interventions, but also prompt us to reconsider the nature of the therapeutic relationship in the digital age. As technology continues to reshape our modes of interaction, ABP stands at the forefront of a new paradigm in mental health care that harnesses the power of the virtual world to foster real-world healing and growth.

## Abbreviations

ABP: avatar-based psychotherapy
